# Aortoenteric Fistula after Endovascular Aneurysm Repair

**DOI:** 10.1155/2021/8828838

**Published:** 2021-02-18

**Authors:** Thilina Gunawardena, Balasubramanium Saseekaran, Sachith Abeywickrama, Rezni Cassim, Mandika Wijeyaratne

**Affiliations:** National Hospital of Colombo, Sri Lanka

## Abstract

Aortoenteric fistula is a rare complication following endovascular abdominal aortic aneurysm repair. However, there is a significant morbidity and mortality associated with this complication. Patients can present with gastrointestinal hemorrhage, fever, or nonspecific features of chronic infection. Extra anatomic bypass with complete graft explanation is the standard management.

## 1. Introduction

Endovascular repair of abdominal aortic aneurysms (AAA) is an alternative to open repair especially in high risk patients. Aortoenteric fistula (AEF) after open AAA repair is a well-known complication but this is rarely reported following endovascular repair. We present a 73-year-old male patient who underwent endovascular repair of an infrarenal AAA, which was complicated with an AEF and graft infection. He underwent axillary-bifemoral bypass and graft explanation but eventually succumbed due to multiple postoperative complications.

## 2. Case

A 73-year-old patient who was on follow-up for diverticulosis was incidentally detected to have an infrarenal AAA with a maximum anteroposterior diameter of 5.5 cm. He was on treatment for type 2 diabetes, hypertension, and hypercholesterolemia. In 2016 July, he underwent endovascular repair of the aneurysm using a bifurcated aortic stent graft system. His recovery was uneventful, and he was discharged 3 days after the procedure. Afterwards, he was lost to follow-up.

In 2019 August, the patient was readmitted to the hospital with severe malaena. No source for acute bleeding was detected on upper and lower GI endoscopy.

In 2019 October, the patient developed fever with chills and rigors and required another hospital admission. Blood investigations showed neutrophil leukocytosis with high CRP and ESR. An exhaustive search for the source of infection was done but all the investigations were negative. Despite broad spectrum antibiotics, the fever persisted, and the inflammatory markers remained elevated.

**Figure 1:**
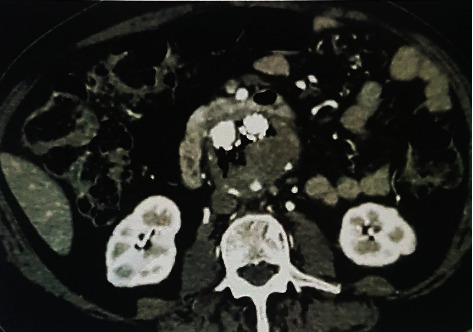
Perigraft gas loculi on CECT.

During the same hospital stay, he developed sudden onset pain and weakness of the left lower limb with absent popliteal and pedal pulses. Acute limb ischaemia (ALI) was suspected, and a contrast CT of the abdomen and a CT angiogram of the lower limbs were done. There was a localized filling defect seen in the left popliteal artery with poor distal run off. The CECT abdomen showed a 5.7 cm size infrarenal AAA with an intraaortic stent graft. There was multiple gas loculi within the sac of the aneurysm (Figure 1). Contrast filling was noted in the posterior aspect of the sac suggestive of a type II endoleak due to a patent lumbar artery. Considering the history of upper GI bleeding and the imaging findings supportive of stent graft infection, a diagnosis of AEF was made.

Initially, he underwent a left popliteal embolectomy to restore limb perfusion. Culture of the retrieved embolus yielded a growth of coliform organisms. This was indicative of septic embolism from the infected aneurysm sac.

**Figure 2:**
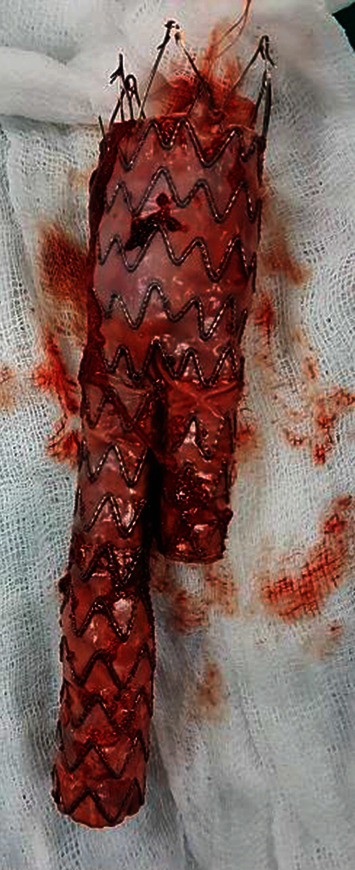
Explanted stent graft.

After 1 week from the embolectomy, he was scheduled for explanation of the infected endograft. First, an axillary-bifemoral bypass was done using the left axillary artery as the inflow vessel, using 6 mm PTFE grafts. A midline laparotomy was done and the neck of the aneurysm defined. A fistulous connection was noted between the 3^rd^ part of the duodenum and the infrarenal aorta. The tract appeared to communicate with the true aortic lumen which was not covered by the endograft. Clamps were placed on bilateral common iliac arteries and the infrarenal aorta. A longitudinal aortotomy was made. The iliac limbs of the graft were pulled out. The hooks of that anchor the proximal graft to the aortic wall were cut using scissors, and the proximal graft was pulled out (Figure 2). The aortic stump and the iliac artery stumps were oversewn. The defect in the duodenal wall was repaired using 3 0 polyglycolic acid suture. Intraoperative blood loss was 5 L. The patient was transferred to the intensive care unit without extubation for postoperative care.

During the ICU stay, he developed acute kidney injury which required continuous renal replacement therapy. It was difficult to wean him off from the ventilator, and he eventually succumbed to ventilator associated pneumonia on postoperative day 12.

## 3. Discussion

An AEF occurring in the setting of a previously untreated aneurysm is considered as a primary fistula while those that occur following aneurysm repair are termed secondary [1]. The exact incidence of secondary AEF after EVAR is unknown as only a few cases have been reported worldwide [2]. The MAEFISTO study reports this risk to be 0.46% when endovascular repair was done as the primary intervention for the AAA. The risk was 3.9%when EVAR was used as a secondary procedure to treat pseudoaneurysms after open repair [3].

## 4. Presentation

Patients with secondary AEF after EVAR often present with nonspecific vague symptoms. Fever, abdominal pain, and gastrointestinal bleeding are the common manifestations [2–4]. Less than <20% present with shock due to hemorrhage as the aneurysm is usually excluded by the previously deployed stent graft [3]. Some patients present with low-grade fever, loss of appetite, and loss of weight due to chronic low grade infection. Septic embolism is another mode of presentation [4].

Timing of AEF after EVAR is variable. It may declare itself as early as after 4 months postprocedure but cases have been reported after 5 years form stent deployment [5]. A high degree of clinical suspicion is required to arrive at the correct diagnosis. As EVAR is a rarely performed in Sri Lanka due to its prohibitive cost, clinicians who initially evaluated our patient did not suspect this complications as the root cause for his symptoms and signs. As a result, the diagnosis was significantly delayed until he developed ALI due to septic embolism.

When a patient presents with upper gastrointestinal (UGI) bleeding endoscopy is the usual first line investigation but a negative study does not rule out a possible AEF. In the MAEFISTO study, 25% of the patients had negative UGI endoscopy findings [3]. However, endoscopy definitely has a role in ruling out other possible causes of UGI bleeding [5].

Contrast CT is the first imaging study done on most of the patients who are suspected with this complication. CT scan clinches the diagnosis in 33-80% of the patients [4, 5]. CT was reported to be 100% sensitive in the MAEFISTO study [3]. Characteristic CT finding is the presence of gas around the graft, periaortic fluid collections, and thickening of the duodenal wall. When CT is inconclusive, FDG-PET scans can be used to detect prosthetic graft infection, albeit with a false positive risk [3].

## 5. Eatiology

Multiple aetiologic mechanisms have been proposed as to how a patient may develop an AEF following EVAR. The most likely possibility is erosion of the aortic wall by the stent graft. Stent migration, fracture, and kink may exert pressure on the aortic wall leading to its erosion. The fixing hooks of the graft can directly damage the bowel wall [4].

Progressive sac expansion due to endoleak or endotension can result in erosion of the aorta and the adjacent bowel. Some authors have considered fistulae occurring by this mechanism as primary AEF. [6] Our patient had the communication between 3^rd^ part of the duodenum and his native aorta which was not covered by the stent graft so theoretically, we can consider this as a primary AEF due to type II endoleak.

AEFs have been reported following coil embolization for endoleaks and erosion of the coils in to the duodenum that has been cited as the possible mechanism in this setting [7]. Fistulation has been reported after endovascular repair of inflammatory aneurysms. Nonspecific periaortic inflammation triggered by the graft itself may lead to AEF formation [8, 9]. AEF after EVAR has been reported in a patient with Crohn's disease, possibly as a result of a diseased small bowel segment eroding in to the aneurysm [10].

## 6. Treatment

Management of AEF following EVAR is challenging, and it has a mortality rate of 50-100%. The complexity of the surgery itself, systemic sepsis, and the poor physical condition in the majority who undergo surgery has been cited as causes for this high mortality. Most of the patients require supracoeliac or suprarenal aortic clamping with a great toll on the physiology [3]. Due to this, some have attempted conservative management in stable patients who are deemed unfit for surgery with long-term antibiotics [3, 11]. However, this strategy is also reported to have a 100% mortality at 1 year [11].

Complete graft explanation with extra anatomic reconstruction is the gold standard of management [4]. Axillary-bifemoral bypass grafting followed by complete removal of infected graft material and over sewing of the aortic and iliac artery stumps should be done. However, some authors describe acceptable success with graft explanation and in situ aortic reconstruction. Antimicrobial impregnated grafts (silver/rifampicin) and cryopreserved allografts have been used as the conduits. The advantages of in situ reconstruction are avoiding additional incisions, elimination of the risk of stump blowout, and prevention of amputation risk due to graft limb occlusion. When the degree of peritoneal contamination is high with purulent material, it is best to avoid in situ reconstruction. [3] The defect in the bowel wall is almost always repaired primarily.

Operated and conservatively managed patients require antibiotics preferably directed by culture sensitivity. A minimum duration of 8 weeks is recommended. Otherwise, antibiotics should be generally continued until clinical and laboratory parameters of infection have subsided. Lifelong antibiotics are recommended in conservatively managed patients, those with incomplete graft explanation and highly virulent organisms [3].

## 7. Conclusions

AEF after EVAR is a rare complication. However, the associated morbidity and mortality are extremely high. Conservative management with long-term antibiotics is an option in stable patients with a frail physiology but the results are disappointing. Operative management with graft explanation, vascular reconstruction with extra anatomic bypass, or in situ graft are the best management options. Patients usually require long-term antibiotics and lifelong close surveillance.
